# P2X7 receptor-dependent tuning of gut epithelial responses to infection

**DOI:** 10.1038/icb.2016.75

**Published:** 2016-09-20

**Authors:** Szu-Wei Huang, Catherine Walker, Joanne Pennock, Kathryn Else, Werner Muller, Michael JD Daniels, Carolina Pellegrini, David Brough, Gloria Lopez-Castejon, Sheena M Cruickshank

**Affiliations:** 1 Faculty of Biology, Medicine and Health, The University of Manchester, Manchester, UK; 2 Department of Clinical and Experimental Medicine, University of Pisa, Pisa, Italy

## Abstract

Infection and injury of the gut are associated with cell damage and release of molecules such as extracellular adenosine 5′-triphosphate (ATP), which is recognised by the purinergic P2X7 receptor (P2X7R). P2X7R is widely expressed in the gut by antigen-presenting cells (APCs) and epithelial cells, but the role of the P2X7R on epithelial cells is poorly understood. We investigated P2X7R in intestinal epithelium *in vitro* and *in vivo* using two model infections, *Toxoplasma gondii* and *Trichinella spiralis*. Lipopolysaccharide and ATP treatment of intestinal epithelial cells and infection with *T. gondii in vitro* did not promote inflammasome-associated interleukin-1β (IL-1β) or IL-18 secretion, but promoted C–C motif chemokine ligand 5 (CCL5), tumour necrosis factor-α and IL-6 production that were significantly reduced when the P2X7R was blocked. Similarly, *in vivo*, infection with either *T. spiralis* or *T. gondii* induced rapid upregulation of epithelial CCL5 in wild-type (wild-type (WT)) mice that was significantly reduced in P2X7R^−/−^ littermate controls. The effects of reduced epithelial CCL5 were assayed by investigating recruitment of dendritic cells (DCs) to the epithelium. Infection induced a rapid recruitment of CD11c^+^CD103^+^ DC subsets into the epithelial layer of WT mice but not P2X7R^−/−^ mice. *In vitro* chemotaxis assays and bone marrow chimeras demonstrated the importance of epithelial P2X7R in DC recruitment. P2X7R signalling in epithelial cells mediates chemokine responses to promote initiation of host immunity to infection.

The gastrointestinal tract is a major route of entry for pathogens and is protected by a variety of defenses, the most important of which is the epithelial lining of the gastrointestinal tract. Luminal antigen recognition and processing begin at the epithelial level leading to orchestration of the coordinated activity of the underlying innate immune cells of the lamina propria including dendritic cells (DCs) and macrophages. As well as responding to the threat of invading pathogens, the gut must also be tolerant to host microbiota. Both host microbiota and pathogens express highly conserved molecular patterns that could potentially trigger host immunity, although most commensal microbiota are noninvasive.^1^ Further, there is also physical separation of host microbiota and the epithelium in the gut because of the presence of a dense mucus layer.^1, 2^ Therefore, a key discriminating feature of a pathogen vs a host microbiota species is that pathogens actively invade and breach the epithelial layer causing cell damage and death.

Following tissue damage and necrotic cell death, irrespective of the initiating stimuli, a number of damage-associated molecular pattern molecules are released. Many damage-associated molecular pattern molecules are cytosolic or nuclear components including adenosine 5′-triphosphate (ATP).^3^ Extracellular ATP is sensed by the ATP-gated purinergic P2X receptors (P2XRs). There are seven different mammalian genes encoding for P2X receptor subunits, P2X1–P2X7, and the P2X7R has a longer C-terminal sequence that is unique among the P2X family.^4, 5^ Activation of P2X7R by ATP induces Ca^2+^ and Na^+^ influx, K^+^ efflux and causes an alteration of cell permeability by causing the formation of a large membrane pore, which eventually leads to cell death.^6^ Under pathophysiological conditions, ATP released from dying cells can enhance P2X7R activation^7^ and trigger activation of the NLRP3 ((NLR family, pyrin domain containing 3)) inflammasome-promoting secretion of the proinflammatory cytokines interleukin-1β (IL-1β) and IL-18.^4^ Although the P2X7R is expressed by a variety of cell types including immune^4^ and epithelial cells,^8^ the activity of P2X7R is best described in immune cells.

P2X7R expression is linked with the development of visceral hypersensitivity in *Trichinella spiralis* (*T. spiralis*) infection.^9^ P2X7R expression in immune cells is also implicated in the control of intracellular infections such as *Toxoplasma gondii* (*T. gondii*). Mutations in P2X7R are associated with enhanced susceptibility to *T. gondii* in humans.^10^ Furthermore, P2X7R in macrophages and DCs promote phagolysosomal fusion and parasite killing *in vitro*.^10, 11^ However, little is known about the role of P2X7R in epithelial cells and the potential role of P2X7R as an epithelial sensor for infection and damage. Previously, we demonstrated that intestinal epithelial cells sense pathogens and initiate host immunity by secreting chemokines that promote rapid recruitment of DCs to the site of infection.^12^ DCs reside in the gut as immature cells and in response to infection or injury are recruited from the gut and also from bone marrow-derived progenitors to the site of infection or injury. After priming they mature and express CCR7, which facilitates migration to secondary lymph nodes for the process of T-cell priming.^13^ Thus, DCs are critical cells acting as the bridge between adaptive and innate immunity. Epithelial cell-derived inflammatory cytokines and chemokines are important for the initiation of gut inflammation and recruitment of inflammatory immune cells such DCs and other cells such as neutrophils.^14^ Our data indicated that a deficient epithelial response is associated with enhanced susceptibility to infection.^12, 15^ Given that the intestinal epithelium is the major entry site of pathogens, and produces infection-induced chemokines and cytokines,^14^ it seems probable that epithelial cells act as a sensor of infection.

In this study, we investigated the role of P2X7R in intestinal epithelial cells in response to two model infections, *T. gondii and T. spiralis*. *In vitro* we found that P2X7R in epithelia promoted chemokine production independently of inflammasome-associated cytokines IL-1β and IL-18. Investigations *in vivo* showed that P2X7R also promoted epithelial chemokine production in response to *T. gondii* and *T. spiralis* infection. Furthermore, reduced epithelial chemokine responses in P2X7R^−/−^ mice were associated with a significant reduction in early infiltration of CD103^+^ DCs to the small intestinal epithelium. Our data indicate a novel role for P2X7R in epithelial cells in the initiation of small intestinal inflammation through chemokine production and recruitment of DCs to the site of infection.

## Results

### P2X7R in epithelial cells promotes CCL5 production

**Figure 1:**
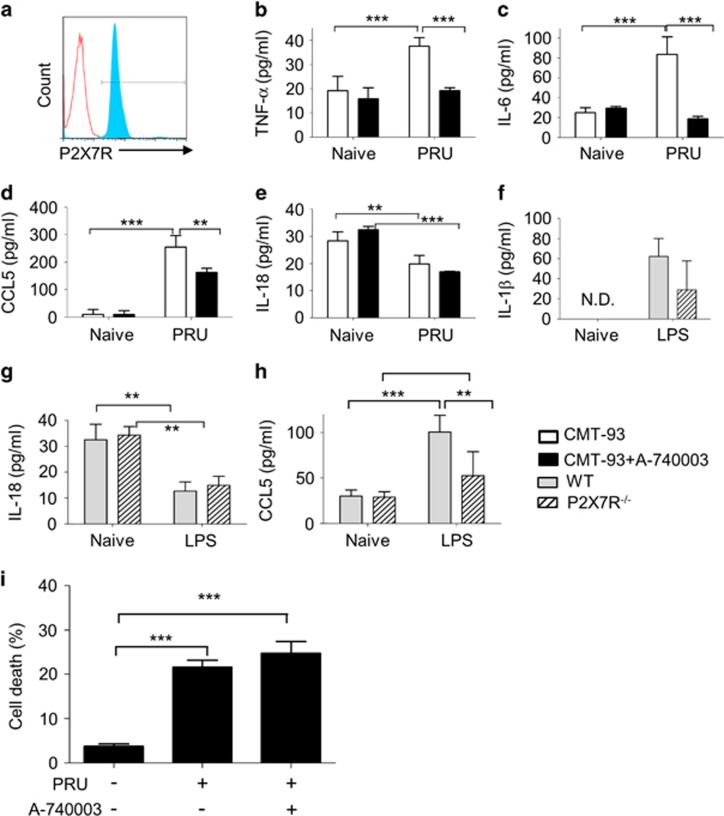
P2X7R promotes chemokine production in primary intestinal epithelial cells. P2X7R expression was confirmed in mouse intestinal epithelial CMT-93 cells by flow cytometry. (**a**) Representative histogram of isotype control stain (red) and CMT-93 cells stained with P2X7R (blue). CMT-93 cells were treated with (closed bars) or without (open bars) the P2X7R inhibitor A-740003 (500 μM) and then infected by *T. gondii* for 24 h (*n*=4). Cell supernatants were analysed by ELISA for TNF-α (**b**), IL-6 (**c**), CCL5 (**d**) and IL-18 (**e**). Small intestinal crypt cells were isolated from C57BL/6 (grey bars) or P2X7R^−/−^ mice (hatched bars), cultured as *ex vivo* crypt organoids and treated with or without LPS (20 μg ml^−1^) for 6 h. Cell supernatants were analysed by ELISA for IL-1β (**f**), IL-18 (**g**) and CCL5 (**h**). (**i**) Following infection by PRU (in a ratio of 1:1) for 24 h, CMT-93 cells with or without the P2X7R antagonist A-740003 treatment (500 μM) were collected for analysis of cell death by Annexin-V/PI (propidium iodide) staining. (**a**) Apoptotic cells were quantified, indicating that A-740003 treatment had no effect on infection-induced cell death. The data are means±s.d. pooled from three independent experiments. ND, not detectable. *P<0.05, **P<0.01 and ***P<0.001. Statistical difference was measured using two-way ANOVA with Bonferroni post-test.

Mouse colonic epithelial CMT-93 cells were infected with *T. gondii* plus or minus a selective P2X7R inhibitor, A-740003, for 24 h. Expression of P2X7R by CMT-93 cells was confirmed by flow cytometry (Figure 1a) and infection of epithelial cells was confirmed by immunohistochemistry and flow cytometry (Supplementary Figure 1). Secretion of ATP by intestinal epithelial cells in response to infection with *T. gondii* was confirmed using an ATP luciferin–luciferase bioluminescence assay (Supplementary Figure 1). Infected epithelial cells produced tumour necrosis factor-α (TNF-α) and IL-6 and the levels of these cytokines were significantly decreased in the presence of the P2X7R inhibitor implicating P2X7R in the production of infection-induced cytokines (Figures 1b and c). As we have previously established that epithelial cells produce C–C motif chemokine ligand 5 (CCL5) in response to infection that drives DC recruitment,^12, 15^ we investigated production of CCL5. Infection with *T. gondii* induced a robust secretion of CCL5, which was significantly reduced by A-740003 (Figure 1d, *P*=0.0058). A-740003 treatment did not alter basal cytokine or chemokine levels (Figure 1). These data suggest that P2X7R was specifically involved in the response to infection. P2X7R stimulation is known to be important for capase-1 activation and the assembly of the inflammasome in antigen-presenting cells.^16^ Therefore, we tested whether P2X7R in epithelial cells also promoted production of the inflammasome-associated cytokines IL-1β and IL-18 in response to *T. gondii* infection. However, we found no detectable IL-1β production by CMT-93 cells (data not shown). IL-18 was secreted at low levels by CMT-93 cells, but this response was not affected by infection or P2X7R inhibition (Figure 1e). In contrast, we assayed the macrophage line THP-1 infected with *T. gondii* and showed they made a robust IL-1β response to infection (Supplementary Figure 2). Additional experiments were performed to contrast the role of P2X7R in epithelial vs macrophage lines. Immortalised bone marrow-derived mouse macrophages and macrophage vs epithelial cells were left unstimulated or stimulated with lipopolysaccharide (LPS) or LPS with ATP and IL-1β secretion was measured. The data showed that there was a significant induction of IL-1β in LPS- and ATP-treated macrophages but not IL-1β secretion by CMT-93 cells (Supplementary Figure 2). The possibility that the inhibitor was having a toxic effect on *T. gondii* was also investigated to ensure the reduction in chemokine production was not due to reduced parasite viability. *T. gondii* were pre-treated with A-740003 for 4 h, washed to remove all the inhibitor and then used to infect CMT-93 cells for 24 h. We found no difference in the percentage of parasite-infected epithelial cells if we used *T. gondii* that had been pre-treated with P2X7R inhibitor (data not shown). These data show that the P2X7R inhibitor is not toxic to the parasite. Collectively, our findings suggest that P2X7R-dependent regulation of chemokines and proinflammatory cytokine production in response to *T. gondii* infection is independent of inflammasome-regulated cytokines.

To investigate the role of the P2X7R further, we used a primary crypt organoid culture model. Small intestinal organoid cells from C57BL/6 (wild-type (WT)) or P2X7R^−/−^ mice were treated with LPS. IL-1β and IL-18 were secreted at very low levels by primary small intestinal epithelial cells and this was not affected by LPS treatment (Figures 1f–h). A lack of P2X7R had no significant effect on IL-1β or IL-18 production in response to LPS, although there was a slight decrease in IL-18 production (Figures 1f and g). P2X7R deficiency did not influence basal levels of CCL5 secretion from crypt organoids (30±6.8 pg ml^−1^ (WT) vs 28±6.0 pg ml^−1^ (P2X7R^−/−^)). However, in agreement with our data using the epithelial cell line, WT organoids secreted significantly more CCL5 than P2X7R^−/−^ organoids in response to LPS stimulation (Figure 1h; *P*=0.046). P2X7R activation is known to induce caspase-1-^17^ and caspase-11-dependent cell death.^18^ As we did not see evidence of epithelial production of the inflammasome-associated cytokines IL-1β and IL-18, we investigated whether the epithelial P2X7R was involved in cell death in *T. gondii* infection. Thus, we analysed the proportion of apoptotic cells in CMT-93 cells infected with *T. gondii* for 24 h using flow cytometry. Compared with naive cells, *T. gondii* infection caused a significant increase in CMT-93 cell death (Figure 1i). However, P2X7R inhibition did not alter the percentage of dead cells in response to infection (Figure 1i), suggesting that the P2X7R was not involved in *T. gondii*-induced epithelial cell death.

### P2X7R in epithelial cells promotes CCL5 production *in vivo*

**Figure 2:**
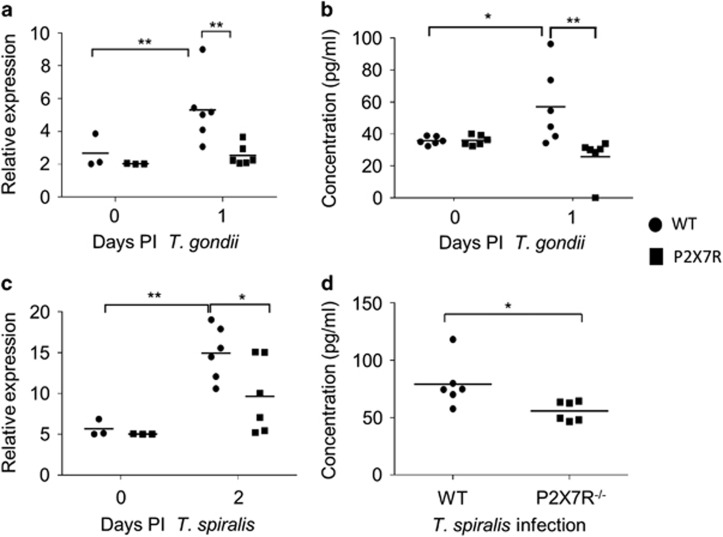
P2X7R promotes epithelial CCL5 production *in vivo* in response to infection. Epithelial cells from the small intestines of C57BL/6 and P2X7R^−/−^ mice at day 0 and 1 p.i. with *T. gondii* (**a** and **b**) and day 0 and 2 p.i. with *T. spiralis* (**c** and **d**) were collected at autopsy and analysed by qPCR for expression of (**a** and **c**) CCL5. (**b** and **d**) Serum CCL5 was analysed by cytokine bead array (CBA) at day 0 and day 1 p.i. with *T. gondii* and day 2 p.i. with *T. spiralis* (C57BL/6 are open bars and P2X7R^−/−^ are closed bars). Data shown are for individual mice (*n*=3–6), with mean value per group, and are pooled from two independent experiments (means±s.d.). *P<0.05 and **P<0.01. Statistical difference was measured using two-way ANOVA with Bonferroni post-test.

To confirm the role of P2X7R in epithelial cell chemokine production *in vivo*, WT and P2X7R^−/−^ mice were infected with *T. gondii*, and chemokine production in intestinal epithelial cells was analysed at days 0 and 1 post-infection (p.i.) by quantitative PCR (qPCR) (Figure 2a). We investigated the expression of CCL5 as we had previously described epithelial cells as a source of this chemokine. Similar to the *in vitro* data CCL5 was upregulated by infection (*P*<0.01; Figure 2a). In contrast, CCL5 expression levels at day 1 p.i. remained low in P2X7R^−/−^ epithelial cells (Figure 2a). We next investigated levels of IL-1β and the results were consistent with our *in vitro* data in that we saw no difference in expression between WT and P2X7R^−/−^ in epithelial cells (data not shown). As the P2X7R has also been associated with production of IL-33, we assayed IL-33 but saw no difference between WT and P2X7R^−/−^ epithelial cells. As there was significantly less early epithelial CCL5 expression in P2X7R^−/−^ mice, the serum chemokine levels of the animals at day 1 p.i. were assessed. Consistent with the qPCR data, P2X7R^−/−^ mice had significantly lower serum CCL5 levels when compared with WT mice (57±9.7 pg ml^−1^ in WT vs 26±5.2 pg ml^−1^ in P2X7R^−/−^) (Figure 2b; *P*<0.01).

To determine whether the altered chemokine response in *T. gondii*-infected P2X7R^−/−^ mice was also true for another small intestinal infection, we also analysed epithelial CCL5 in *T. spiralis* infection. Similar to the *T. gondii* data, both WT and P2X7R^−/−^ mice had increased expression of epithelial CCL5 relative to naive mice following infection (Figure 2c) and CCL5 expression was significantly reduced in P2X7R^−/−^ epithelial cells (Figure 2c; 10±3.3 in WT vs 4.5±4.0 in P2X7R^−/−^, *P*=0.042). Consistent with the qPCR data, P2X7R^−/−^ mice had significantly lower serum CCL5 levels in response to *T. spiralis* when compared with WT mice (79±21 pg ml^−1^ in WT vs 55±8.5 pg ml^−1^ in P2X7R^−/−^) (Figure 2d, *P*=0.03). Collectively, these data confirm our *in vitro* data that P2X7R deficiency impairs the epithelial chemokine response to infection.

As *T. gondii* causes damage to the gut and *T. spiralis* also burrows into intestinal epithelial cells causing damage and intestinal epithelia cells express low levels of P2X7R,^19^ we investigated the role of epithelial P2X7R in the apoptotic response to infection. The analysis of ileum sections using TUNEL (terminal deoxynucleotidyl transferase dUTP nick-end labelling) assay showed that P2X7R^−/−^ mice had significantly less cell death in response to infection as compared with WT mice (Supplementary Figure 3). This is in line with published work showing that P2X7R promotes cell death, but as P2X7R is expressed by multiple cell types in the gut, it is not possible to ascribe whether P2X7R expressed in the epithelium is driving cell death. Collectively, however, these data imply a role for epithelial P2X7R in the early response to infection-induced cellular damage.

### Impaired recruitment of CD103^+^ DC subsets to the small intestinal epithelium in infected P2X7R^−/−^ mice

**Figure 3:**
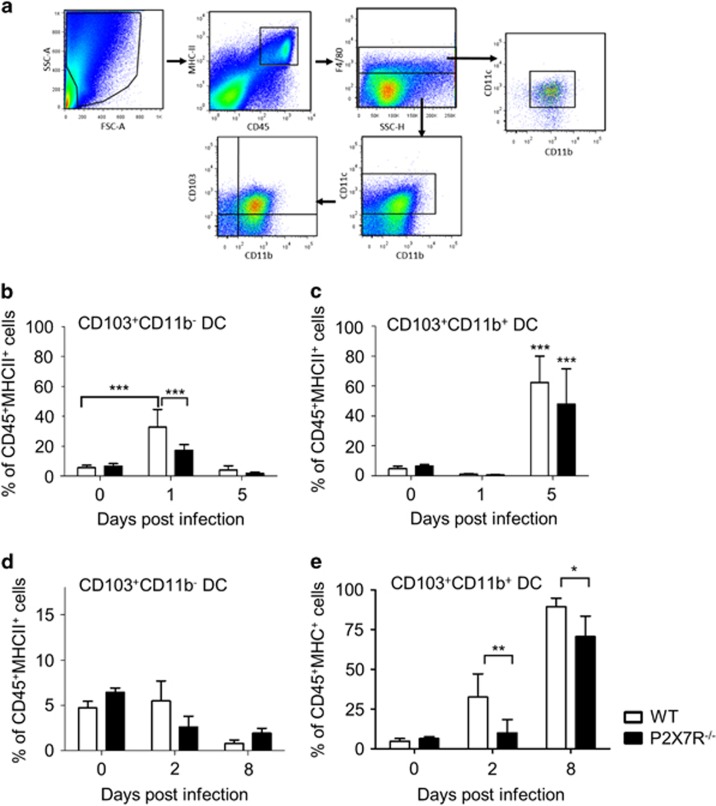
Impaired early recruitment of CD11c^+^CD103^+^CD11b^−^ DCs in P2X7R^−/−^ mice after infection. C57BL/6 and P2X7R^−/−^ mice were orally infected with *T. gondii* and killed at day 0, 1 and 5 p.i. or *T. spiralis* and killed at day 0, 2 and 8 p.i. (**a**) Gating strategy for distinction between small intestinal CD11c^+^ MHCII^hi^ subsets identified as F4/80^−^CD103^+^CD11b^−^ DC (termed CD103^+^CD11b^−^ DC), F4/80^−^CD103^+^CD11b^+^ DC (termed CD103^+^CD11b^+^ DC), CD103^−^CD11b^+^ DC and CD11c^+^F4/80^+^ macrophages. The frequency of intraepithelial CD103^+^CD11b^−^ DC (**b** and **d**) and CD103^+^CD11b^+^ DC (**c** and **e**) were calculated as the percentage of CD45^+^MHCII^+^ cell population in C57BL/6 (open bars) and P2X7R^−/−^ mice (closed bars); *n*=6 per group from two experiments (mean±s.d.). *P<0.05, **P<0.01 and ***P<0.001. Statistical difference was measured using two-way ANOVA with Bonferroni post-test.

**Figure 4:**
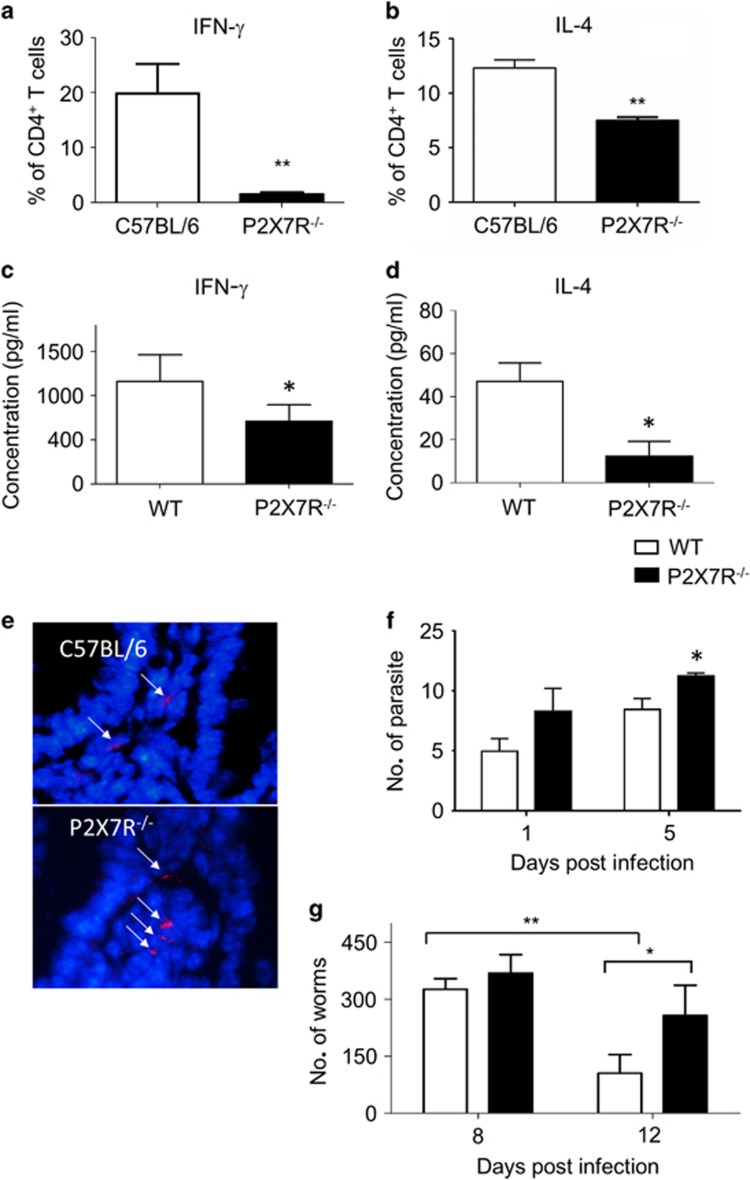
Impaired adaptive immunity to infection in the absence of P2X7R. Splenocytes from WT and P2X7R^−/−^ mice day 8 p.i. with *T. gondii* (**a**) or *T. spiralis* (**b**) were analysed by flow cytometry for the percentage of CD4^+^ cells from WT (white bars) and P2X7R^−/−^ mice (black bars) expressing IFN-γ (**a**) or IL-4 (**b**). Serum IFN-γ (**c**) was measured from *T. gondii*-infected mice. Supernatants from *Trichinella* antigen-stimulated WT and P2X7R^−/−^ splenocytes taken at day 8 p.i. were collected for analysis of (**d**) IL-4. Parasite load was confirmed by counting the tomato-red-expressing *T. gondii* parasites in the ileum by microscopy. (**e**) Representative images of ileum with red *T. gondii* parasites (arrows) and a DAPI (4',6-diamidino-2-phenylindole) (blue) counterstain and (**f**) quantification of parasite load per field of view in C57BL/6 (open bars) and P2X7R^−/−^ (black bars) mice (scale bar: 20 μm). (**g**) Worm burdens at day 8 and 12 p.i. were quantified. Data shown are means±s.d. pooled from two independent experiments. *P<0.05 and **P<0.01.

We have previously shown that epithelial chemokine production promotes DC recruitment to the gut during infection.^12, 15^ To assess whether P2X7R signalling has a role in recruitment of DCs to the intestinal epithelium, we investigated DCs and macrophages in the small intestine following *T. gondii* and *T. spiralis* infection (Figure 3 and Supplementary Figure 4). Cells were first gated based on whether they were alive, doublets were excluded and the live CD45^+^CD11c^+^ MHCII^hi^ population selected for subsequent analysis of DC subsets. The percentages of CD103^+^ DC subtypes in intestinal epithelial layers were similar between naive WT (CD103^+^CD11b^−^ DC: 5.3±1.6% and CD103^+^CD11b^+^ DC: 5.8±4% of CD45^+^ population) and naive P2X7R^−/−^ (CD103^+^CD11b^−^ DC: 5.8±1.5% and CD103^+^CD11b^+^ DC: 6.8±0.4% of CD45^+^ population) mice (Figures 3b–e). At day 1 p.i. with *T. gondii*, WT mice had a sixfold increase in the proportion of CD103^+^CD11b^−^ DCs (32.68±4.9%) in the epithelial layer, compared with P2X7R^−/−^ animals in which there was only a threefold increase (17.28±1.7%, *P*<0.001) (Figure 3b). By day 5 p.i., the frequency of epithelial-associated CD103^+^CD11b^−^ DCs were similar to naive cells in both WT and P2X7R^−/−^ animals, whereas the intraepithelial CD103^+^CD11b^+^ DCs in WT and P2X7R^−/−^ mice increased albeit to a similar magnitude (Figure 4c). In contrast to infection with *T. gondii*, the proportion of CD103^+^CD11b^−^ DCs in the epithelial layer remained constant after infection with *T. spiralis*. However, at day 2 p.i., WT mice had a sixfold increase in the proportion of CD103^+^CD11b^+^ DCs in the epithelial layer (Figure 3d) as well as a threefold increase in lamina propria cells (LPL) (Supplementary Figure 4), compared with P2X7R^−/−^ animals in which the proportion of CD103^+^CD11b^+^ DCs remained low and similar to day 0 (epithelial layer: 32.5±14.7% in WT vs 10±8.5% in P2X7R^−/−^, *P*=0.0088; LPL: 31.2±7.0% in WT vs 16.6±9.0% in P2X7R^−/−^, *P*=0.0121). *T. spiralis*-infected WT and P2X7R^−/−^ mice had an increase in the epithelial layer and LPL CD103^+^CD11b^+^ DCs at day 8 p.i., but the proportion of epithelial-associated CD103^+^CD11b^+^ DCs in the WT epithelial layer was significantly higher compared with P2X7R^−/−^ mice (89.4±5.3% in WT vs 70.6±12.8% in P2X7R^−/−^, *P*<0.05).

There was not a general reduction of DC recruitment into the epithelial layer of the P2X7R^−/−^ intestine. The proportion of CD103^−^ DCs (CD11c^+^CD11b^+^CD103^−^F4/80^−^) were unchanged between WT and P2X7R^−/−^ mice in the epithelial layer or LPL compartment after either *T. gondii* or *T. spiralis* infection (data not shown). Furthermore, the proportions of F4/80^+^CD11c^+^ macrophages were similar between WT and P2X7R^−/−^ before and after infection irrespective of the type of infection (Supplementary Figures 4C,D,G and H). There were no significant differences in the absolute number of CD45^+^ cells between genotypes at all time-points studied. Collectively, these data show that irrespective of the infection, there is a selective reduction in the early recruitment of DC subsets in the absence of P2X7R.

### P2X7R^−/−^ mice have reduced parasite specific T-cell responses and higher parasite burdens p.i.

To assess whether the delayed recruitment of CD103^+^ DCs in response to infection influenced the development of T-cell responses, splenocytes were collected from WT and P2X7R^−/−^ mice at day 8 p.i. and the T-cell response to infection was analysed. For *T. gondii* infection, splenic CD4^+^ T cells were assessed for intracellular cytokine production of interferon-γ (IFN-γ) (Figure 4a). There was a significant increase in the proportion of IFN-γ^+^ cells in WT mice compared with P2X7R^−/−^ (Figure 4a; *P*<0.001). In response to *Trichinella* infection, we saw a significant reduction of IL-4^+^-secreting cells in P2X7R^−/−^ mice compared with WT mice (*P*<0.001; Figure 4b). To confirm whether the reduced T-cell cytokine productions altered the adaptive immune response systemically, serum cytokine levels during infection were analysed. P2X7R^−/−^ mice had significantly less IFN-γ in their serum following *T. gondii* infection compared with control mice at day 8 p.i. (Figure 4c; *P*<0.05), consistent with the reduction in the splenic IFN-γ^+^ CD4^+^ T-cell population. Splenocytes from *T. spiralis-*infected P2X7R^−/−^ mice also produced significantly less IL-4 following *T. spiralis* infection compared with control mice at day 8 p.i. (Figure 4d; *P*<0.05), consistent with the reduction in the splenic IL-4^+^ CD4^+^ T-cell population.

The observation that there was a reduced immune response in P2X7R^−/−^ mice suggested that parasite burden and pathology may also be altered. Therefore, parasite burden in the ileum villi was enumerated. Compared with WT mice, P2X7R^−/−^ mice had significantly higher *T. gondii* tachyzoite burden at day 5 p.i. (*P*<0.05) (Figures 4e and f). Similarly, P2X7R^−/−^ mice had a significantly higher worm burden (*P*<0.05) at day 12 p.i. (Figure 4g). These data implicate a role for P2X7R in controlling the local infection of *T. gondii* and expulsion of *T. spiralis.*

### P2X7R on epithelial cells promotes DC recruitment to the site of infection

**Figure 5:**
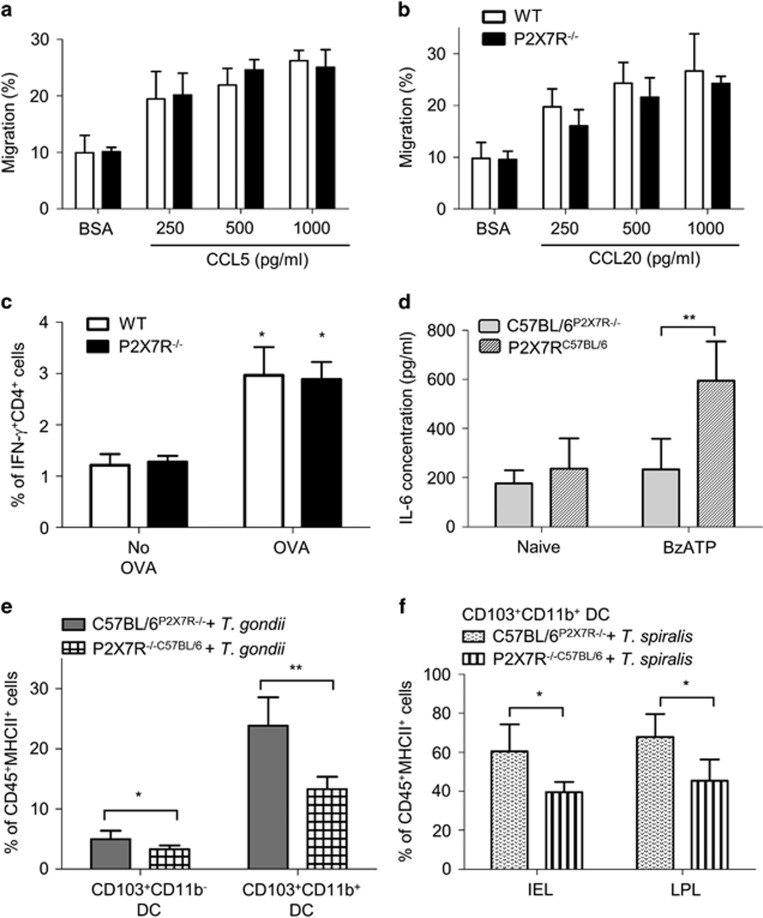
Epithelial P2X7Rs are necessary for the intraepithelial recruitment of DCs. BMDCs from C57BL/6 (open bars) and P2X7R^−/−^ (closed bars) mice were stimulated with (**a**) CCL5 or (**b**) CCL20 and migration was assessed using a transwell migration assay. (**c**) OT-II splenocytes stimulated with OVA-pulsed BMDCs from C57BL/6 (open bars) or P2X7R^−/−^ (closed bars) mice for 48 h, were analysed by flow cytometry for intracellular IFN-γ and quantified by flow cytometry. *P<0.05 and **P<0.01 as compared with BSA treatment (means±s.d., one-way ANOVA with Tukey's test) or no OVA group (means±s.d., two-way ANOVA with Bonferroni post-test). (**d**) Bone marrow chimeras C57BL/6^P2X7R−/−^ (white bars) and P2X7R^−/−C57BL/6^ (black bars) were generated and the chimerism was confirmed by stimulating splenocytes with 3′-O-4-benzoyl-ATP (BzATP) (100 μM) and IL-6 secretion assessed using ELISA. **P<0.01 (means±s.d., two-way ANOVA with Bonferroni post-test). The chimeras were orally infected with *T. gondii* PRU (1 × 10^6^ tachyzoites per mouse) or *T. spiralis* (400 larva per mouse) and killed at day 1 or 2 p.i., respectively. Intraepithelial cells were isolated from the small intestine and stained for CD45, MHCII, F4/80, CD11b, CD11c and CD103. (**e**) The graph shows the percentage of CD11c^+^CD103^+^CD11b^−^ and CD11c^+^CD103^+^CD11b^+^ DCs of the CD45^+^MHC^+^ cells (*n*=6) with mean value per group, and are pooled from two independent experiments (means±s.d.). *P<0.05 and **P<0.01. Statistical difference was measured using Student's *t*-test. (**f**) The percentage of CD11c^+^CD103^+^CD11b^+^ DCs out of the CD45^+^MHCII^+^ cells with means±s.d. (*n*=5 per group, pooled from two different experiments). *P<0.05 and **P<0.01 (Student's *t*-test).

P2X7R is expressed by DCs and epithelial cells; therefore, our KO mice lack functional P2X7R in antigen-presenting cells and epithelial cells. To dissect whether the reduction of early CD103^+^ DC infiltration to the small intestine in P2X7R^−/−^ mice was because of a defect in the capacity of P2X7R^−/−^ DC to migrate in response to chemokines, we generated bone marrow-derived DCs (BMDCs) from WT and P2X7R^−/−^ mice and monitored BMDC migration in response to CCL5 or CCL20, chemokines previously reported to promote DC recruitment to the epithelium.^12, 15^ Both CCL5 and CCL20 induced a two- to threefold increase of BMDC migration and there was no difference of the migratory response between WT and P2X7R^−/−^ BMDCs (Figures 5a and b).

The possibility that P2X7R^−/−^ DCs might not be able to effectively present antigen was next investigated. WT or P2X7R^−/−^ BMDCs that had been pulsed with ovalbumin (OVA) were cocultured with OVA peptide-specific OT-II splenocytes at a ratio of 1:10 for 48 h. Data showed that both WT and P2X7R^−/−^ BMDCs were able to present OVA antigen and induced the development of IFN-γ^+^ CD4 T cells (2.9±0.5% in WT vs 2.8±0.3% in P2X7R^−/−^) to equivalent levels (Figure 5c). Overall, these data indicate that P2X7R^−/−^ DCs are able to respond normally to chemokines and there was no difference in antigen presentation between WT and P2X7R^−/−^ BMDCs.

### Impaired chemotactic function in P2X7R^−/−^ intestinal epithelial cells

As the *in vitro* migration function of P2X7R^−/−^ DCs was normal, we hypothesised that the defect in DC migration *in vivo* was because of the impaired epithelial chemokine responses. To investigate this possibility, we generated bone marrow chimeras. WT and P2X7R^−/−^ mice were irradiated and WT mice were reconstituted with P2X7R^−/−^ bone marrow and P2X7R^−/−^ mice with WT bone marrow (referred to as C57BL/6^P2X7R−/−^ and P2X7R^−/−C57BL/6^). Successful reconstitution was confirmed by PCR of gut cells and also by analysing IL-6 secretion from P2X7R agonist, 3'-O-4-benzoyl-ATP-stimulated splenocytes (Figure 5d); however, we cannot exclude the possibility that some radioresistant cells remained. The P2X7R^−/−C57BL/6^ splenocytes produced significantly higher levels of IL-6 in response to 3'-O-4-benzoyl-ATP treatment as compared with the C57BL/6^P2X7R−/−^ cells (596±158 pg ml^−1^ in P2X7R^−/−C57BL/6^ vs 235±125 pg ml^−1^ in C57BL/6^P2X7R−/−^, *P*<0.01), which is consistent with previous studies.^20^ Following infection, we assessed CD103^+^ DC recruitment to the small intestinal epithelium in chimeric mice. WT mice that were reconstituted with P2X7R^−/−^ bone marrow (C57BL/6^P2X7R−/−^) responded robustly to *T. gondii* infection with DC recruitment into the epithelial layer. In contrast, the P2X7R^−/−^ mice (i.e., those with P2X7R-null epithelium) that had been reconstituted with WT bone marrow (P2X7R^−/−C57BL/6^) had a significant reduction in the magnitude of the CD103 DC response with CD103 DC subsets significantly reduced in the epithelial layer of P2X7R^−/−C57BL/6^ mice (CD103^+^CD11b^−^ DCs: 11.5±0.8% in C57BL/6^P2X7R−/−^ vs CD103^+^CD11b^−^ DCs: 7.6±0.8% in P2X7R^−/−C57BL/6^, *P*=0.0092; CD103^+^CD11b^+^ DCs: 15.5±0.8% in C57BL/6^P2X7R−/−^ vs CD103^+^CD11b^+^ DCs: 8.3±0.6% in P2X7R^−/−C57BL/6^, *P*=0.0001) (Figure 5e). Similarly, at day 2 p.i. with *T. spiralis*, there was a significant reduction in the magnitude of the DC response in both epithelial and LPL compartments in P2X7R^−/−C57BL/6^ compared with C57BL/6^P2X7R−/−^ mice (Figure 5f; epithelial layer CD103^+^CD11b^+^ DCs: 60.5±14% in C57BL/6^P2X7R−/−^ vs 40±5.1% in P2X7R^−/−C57BL/6^, *P*=0.013; LPL CD103^+^CD11b^+^ DCs: 68±11% in C57BL/6^P2X7R−/−^ vs CD103^+^CD11b^+^ DCs: 45.5±11% in P2X7R^−/−C57BL/6^, *P*=0.014). Control experiments were performed where WT and P2X7R^−/−^ mice were also infected by *T. spiralis* and the proportions of CD103^+^CD11b^+^ DCs at day 2 p.i. were analysed and showed the expected reduction in DC recruitment in P2X7R^−/−^ mice (epithelial-associated CD103^+^CD11b^+^ DCs: 26.7±3.7% in C57BL/6 vs 17.6±0.9% in P2X7R^−/−^, *P*=0.0403) (data not shown). Thus, these data indicate that the *in vivo* migration ability of P2X7R^−/−^ DCs to the epithelium is normal in the gut and implicate epithelial P2X7R in mediating the DC recruitment in response to infection-induced injury.

Taken together, our data indicate a role for epithelial P2X7R-sensing infection and producing chemokines that promote the recruitment of immune cells needed for the initiation of effector immunity.

## Discussion

Pattern recognition receptors recognise the highly conserved molecular patterns present on pathogens and also commensal organisms. However, most commensals are not invasive. Therefore, a feature of infection is the damage caused, which is characterised by the release of damage-associated molecular pattern molecules such as ATP. Extracellular ATP is a danger signal that alerts the immune system of abnormal death and threat. Our *in vivo* data indicated that the P2X7R on epithelial cells has a critical role in sensing infection-induced damage. Activation of P2X7R in macrophages and DCs has been linked to activation of the inflammasome. The inflammasome serves as a platform for the activation of caspase-1-mediated cleavage and maturation of IL-1β and IL-18. However, *in vitro* infection or treatment of intestinal epithelial cells by *T. gondii* or LPS did not trigger an IL-1β or IL-18 response regardless of whether the P2X7R was present, suggesting that in epithelial cells P2X7R may have an alternative role. Our data implicated P2X7R in promoting the production of cytokines and chemokines from intestinal epithelial cells. Among these epithelial-derived C–C chemokines, we have shown that CCL2, CCL5 and CCL20 are potential chemoattractants for intestinal DC homing to the site of infection.^12, 15^ Our *in vivo* and *in vitro* data show that the absence of P2X7R results in impaired CCL5 production. Multiple TLRs are involved in sensing *T. gondii* infection and initiating proinflammatory responses. TLR11/12 are required for recognising toxoplasma profilin to regulate DC-derived IL-12 in response to *T. gondii* infection.^21, 22^ TLR2 and TLR-adaptor MyD88^−/−^ mice are more susceptible to *T. gondii* with decreased production of chemokines.^21, 23, 24^ Furthermore, TLRs are known to trigger NF-κB-dependent production of cytokines (e.g., IL-1β, IL-6, TNF-α and type-I interferons)^25^ and chemokines, including CXCL10, CCL2 and CCL5,^26^ to elicit inflammation in response to infection. Thus, to study whether TLR signalling is involved in the P2X7R-dependent CCL5 response, we used LPS, known to induce CCL5 expression in rodent epithelial cells, to stimulate TLR4 in primary crypt organoids. We found that LPS-induced CCL5 production in primary organoids and that the CCL5 upregulation was reduced in the absence of P2X7R. Our *ex vivo* data showed that CCL5 production in response to LPS treatment of WT organoids was also lower in P2X7R^−/−^ organoids. These data suggest that blocking P2X7R signalling attenuates TLR activation-induced chemokine production. Our observation that in the absence of the P2X7R, epithelial responses to infection *in vivo* and *in vitro* were also reduced suggest that the P2X7R may have a regulatory role in pathogen-pattern recognition receptor-mediated proinflammatory signalling.

CCL5 has been shown to drive the recruitment of several cell types including T cells, macrophages, eosinophils and basophils.^27^ Our previous work implicated epithelial chemokine responses in promoting the recruitment of DCs to the site of infection; therefore, we focused on DC recruitment as an indicator of the biological effect of reduced epithelial chemokine signalling. However, it may be that other cells are similarly affected.^12^ The reduction of epithelial chemokine production in P2X7R^−/−^ mice was associated with delayed infiltration of CD103^+^ DCs in response to *T. gondii* and *T. spiralis* infection. Early DC responses are known to alter disease outcome to intracellular pathogen infections including *T. gondii* and *Listeria monocytogenes*.^28, 29^ Intestinal CD103^+^ DCs can be divided into two main subsets, CD103^+^CD11b^+^ and CD103^+^CD11b^−^ DCs, which differ in terms of distribution, requirement for transcription factors and *in vivo* function.^30^ CD103^+^CD11b^−^ DCs are dominant in colon lamina propria and extra-intestinal tissue,^31^ whereas CD103^+^CD11b^+^ DCs are the major subset in the small intestinal lamina propria and villus.^30^ Additionally, the CD103^+^CD11b^−^(CD8α^+^) DC subset in the small intestine expresses several different TLRs and promotes Th1 immunity and CTL activity.^32^ In the *T. gondii*-infected mouse ileitis model, we noted that blocking P2X7R delayed the recruitment of CD103^+^CD11b^−^ DCs, whereas CD103 CD11b^+^ DCs were reduced in *T. spiralis* infection. CD103^+^CD11b^−^ DCs are thought to be the major IL-12-producing immune cell against acute *T. gondii* infection^33^ and have an important role in cross-presentation.^34^ Thus, the intraepithelial recruitment of CD103^+^CD11b^−^ DCs observed in WT mice could be important for early control of *T. gondii* infection. Indeed, our observations that there was a higher parasite load in P2X7R mice could corroborate this, although the altered recruitment of other innate cells such as neutrophils may contribute to this also. The *in vivo* role of CD103^+^CD11b^+^ DCs in small intestinal inflammation is not yet clear, but they are thought to be critical in both tolerogenic and inflammatory responses,^33, 35^ and selective reduction of CD103^+^CD11b^+^ DCs by depleting interferon regulatory factor 4 has been shown to impair Th17 differentiation in mesenteric lymph node.^36^ Thus, it is still unclear which CD103^+^ DC subset has a more important role against Th1 or Th2 infections. A previous report showed that defective chemoattraction of small intestinal CD103^+^ DCs in neonatal mice impairs the development of protective immunity against *Cryptosporidium parvum* infection.^37^ Thus, the delayed chemoattraction of CD103^+^ DC subsets may delay the infiltration of CD103^+^ DCs into the draining lymph nodes for T-cell priming and correlate with the impaired Th immunity seen in P2X7R^−/−^ mice.

Collectively, our findings identify a novel role for small intestinal epithelial P2X7R in the induction of epithelia cytokine responses and early DC infiltration in response to infection. Given the proinflammatory role as a potential tissue damage sensor to initiate inflammation, P2X7R is likely to be an important target for the development of new therapies for inflammatory disorders in the gut.

## Methods

### Mice

C57BL/6 (WT) and P2X7R^−/−^ littermate controls were generated from breeding heterozygous Pfizer P2X7R^−/−^ mice (The Jackson Lab, Bar Harbor, ME, USA).^20^ All mice were maintained by the Biological Services Facility (University of Manchester, UK), and kept in individually ventilated cages and fed a standard chow. Experiments were performed in accordance with the Home Office Animals (Scientific Procedures) Act (1986). Three to six mice per group were used per study and each infection was repeated two to three times. For *T. gondii* infection, age-matched male mice each mouse was orally infected with 1 × 10^6^ of tachyzoites in 0.1 ml phosphate-buffered saline (PBS) in the morning. The maintenance, recovery and infection of *T. spiralis* was described previously.^38^ Age-matched male mice were infected with 400 larvae by oral gavage. Worm burden was assessed by longitudinal section of the small intestine followed by incubation in PBS at 37 °C for 4 h. Mice were monitored throughout infection and no unexpected adverse effects were observed.

### *T. gondii* and cells

*T. gondii* type-I RH strain expressing yellow fluorescence protein^39^ and type-II Prugniaud (PRU) expressing tandem dimers of tomato-red fluorescent protein *T. gondii* strain was used in our experiments.^40^ The tachyzoites were harvested by serial 4–5 days passage in human foreskin fibroblast cultured in Dulbecco's modified Eagle's medium (Sigma-Aldrich, Dorset, UK) with 10% fetal bovine serum (FBS) (Sigma-Aldrich) and 1% penicillin/streptomycin (Sigma-Aldrich). The mouse colonic epithelial cell line, CMT-93, was maintained in Dulbecco's modified Eagle's medium (Sigma-Aldrich) with 10% FBS (Sigma-Aldrich), 2 mM l-glutamine (Sigma-Aldrich) and 1% penicillin/streptomycin (Sigma-Aldrich). CMT-93 cells were infected with PRU at a ratio of 1:5 for 24 h and treated with the selective P2X7R antagonist, A-740003 (500 μM; Sigma-Aldrich) to block P2X7R at the time of infection. A total of 1 × 10^6^ cells per group cells were infected by *T. gondii* PRU strain in a ratio of 1:1. THP-1 monocyte cells were primed using 0.1 μg ml^−1^ of LPS for 24 h followed by infection with *T. gondii* for 24 h in a ratio of 1:4.

Immortalised murine bone marrow-derived macrophages were obtained from Prof Claire Bryant (Department of Veterinary Medicine, University of Cambridge, Cambridge, UK). Immortalised bone marrow-derived mouse macrophages and CMT-93 epithelial cells were cultured in Dulbecco's modified Eagle's medium, 10% FBS (Life Technologies, Paisley, UK), 100 U ml^−1^ penicillin and 100 μg ml^−1^ streptomycin (PenStrep, Sigma, London, UK). Cells were seeded overnight at 5 × 10^5^ cells per ml (immortalised bone marrow-derived mouse macrophages) in 24-well plates. Cells were primed with LPS (1 μg ml^−1^, 2 h). Following priming, inflammasomes were stimulated by adding ATP (5 mM) for 1 h. Supernatants were removed and analysed for IL-1β content by ELISA (DuoSet; R&D Systems, Abingdon, UK) according to the manufacturer's instructions.

### Isolation of small intestinal cells

Cells from the small intestine were isolated as described previously.^41^ Briefly, Peyer's patches were removed, and the remaining tissue cut into pieces and transferred into Hanks' balanced salt solution supplemented with 1% HEPES and 2 mM ethylenediaminetetraacetic acid. The tissue pieces were incubated at 37 °C with shaking for three sets of 15 min and the cells collected and pooled. Intestinal epithelial cells were isolated by Percoll density gradient isolation (Scientific Laboratories Supplies, Yorkshire, UK) and resuspended in RPMI-1640 (Sigma-Aldrich) with 10% FBS and 1% HEPES. The remaining tissue fragments were incubated in RPMI media supplemented with collagenase VIII and CaCl_2_ at 37 °C for 60 min to collect LPL. All the epithelial layer cells and LPL were resuspended at 1 × 10^7^ cells per ml in FACS buffer (PBS plus 2% FBS with 0.1% sodium azide) for flow cytometry analysis.

### Stimulation of cells for cytokine staining

OT-II splenocytes (10^6^ cells per ml) from C57BL/6 OT-II mice with IL-10-green fluorescence protein reporter122 (RAG-1-OT-II × Vert-X F1 cross) were cocultured with OVA-pulsed BMDCs from WT or P2X7R^−/−^ mice at a ratio of 10:1 (BD Falcon, Oxford, UK). After 72 h, the splenocytes harvested were stimulated with phorbol 12-myristate 13-acetate (5 ng ml^−1^; Sigma-Aldrich) and ionomycin (500 ng ml^−1^; Sigma-Aldrich) for 1 h followed by another 3 h with brefeldin A (1 μg ml^−1^; Becton Dickinson (BD), Oxford, UK). For parasite-infected mice, cells from the spleen and mesenteric lymph node were harvested at day 8 p.i. and stimulated following the same protocol as OVA-stimulated OT-II splenocytes. After stimulation, these cells were ready for cell-marker and intracellular cytokine staining.

### Flow cytometry analysis

Cells were incubated with an Fc receptor blocker, anti-CD16/32 antibody (2 μg ml^−1^; eBioscience, Cheshire, UK). After washing, cells were incubated with FITC-anti-CD45 (eBioscience), Pacific Blue-anti-MHCII (BioLegend, London, UK), APC-anti-F4/80 (eBioscience), Alex Fluor 700-anti-CD11c (eBioscience), APC-Cy7-anti-11b (BD) and PerCP/Cy5.5-anti-CD103 (BioLegend) antibodies. For assessing intracellular cytokine expression, cells were labelled with FITC-anti-CD3 (BD) and PerCP-anti-CD4 (BD) antibodies, fixed and permeabilised using FOXP3 Fix/Perm buffer set (BioLegend) followed by staining with PE/Cy7-anti-CD25, Alexa Fluor 647-anti-FOXP3, BV-421-anti-IFN-γ, PE-IL-4 and BV-605 anti-IL-17 antibodies (all from BioLegend). Cells were acquired by flow cytometry on the BD LSRII (BD, Oxford, UK) and the data were analysed using FlowJo flow cytometry software (Tree Star, Ashland, OR, USA). The expression of P2X7R in CMT-93 cells was assessed by intracellular staining of P2X7R using FITC-conjugated rat anti-mouse P2X7R antibody (Aviva System Biology, San Diego, CA, USA) and FITC-conjugated rat IgG1 (BD) was used as isotype control.

### ELISA and cytokine analysis

IL-1β, IL-18 and CCL5 levels in mouse serum and supernatants from CMT-93 and crypt organoids were measured using ELISA DuoSet Kits (R&D Systems) according to the manufacturer's instructions. ATP was measured using the Luciferin–Luciferase Bioluminescence Assay (Thermo Fisher Scientific, Loughborough, UK) as per the manufacturer's instructions on three technical replicates. Levels of IL-4, IL-10, IL-6, IL-9, IL-13, IFN-γ, TNF-α and IL-12p70 in the serum were determined using a cytometric bead array according to the manufacturer's instructions (BD) and analysed using BD FacsAria cytometer (BD) and FCAP Array software (BD).

### Mouse bone marrow-derived DCs

Bone marrow cells from C57BL/6 and P2X7R^−/−^ mice were cultured in BMDC culture media containing RPMI-1640 (Sigma-Aldrich) with 10% FBS (Thermo Fisher Scientific), 1% penicillin/streptomycin, 1% l-glutamine, 50 μM β-mercaptoethanol and 4% granulocyte–macrophage colony-stimulating factor. After 6 days of culture, the phenotype of BMDCs was confirmed by staining with PE-Cy7-anti-CD45, Alexa Fluor 700-anti-CD11c (eBioscience) and Pacific Blue-anti-MHCII (BioLegend) antibodies, and the purity of MHCII^+^CD45^+^CD11c^+^ BMDCs was >90%. Splenocytes (10^6^ cells per ml) from C57BL/6 OT-II mice were cocultured with OVA-pulsed BMDCs from WT or P2X7R^−/−^ mice at a ratio of 10:1 in a 48-well plate (BD Falcon) for 72 h.

### Chemokine migration assay

BMDCs from C57BL/6 or P2X7R^−/−^ mice were added to the upper well of transwell plates (Fisher Scientific, Loughborough, UK) at 1 × 10^6^ per well and 0.1% bovine serum albumin, CCL5 or CCL20 chemokine (both from R&D Systems) added to the bottom well in a twofold serial dilution (1000–250 pg ml^−1^). After incubating for 3 h at 37 °C, the number of cells in the bottom well were counted in triplicate using a CASY TT Cell Counter (Roche Innovatis AG, Bielefeld, Germany). The percentage of migrated cells was calculated by subtracting background migration from the bovine serum albumin only data.

### Small intestinal epithelial cell isolation and qPCR

Pieces of small intestine tissue were incubated in Hanks' balanced salt solution supplemented with 1% HEPES and 2 mM ethylenediaminetetraacetic acid at 37 °C with shaking for 30 min and the epithelial cells collected. Epithelial purity was confirmed by flow cytometry using CD326 (Ep-CAM; Cambridge Bioscience, Cambridge, UK) and CD45-FITC (eBioscience) and preparations were >95% pure. Total RNA was isolated from cells by homogenising in TRIsure (Bioline, London, UK) and chloroform/isopropanol isolation (Sigma-Aldrich) and the concentration of RNA measured on a Nanodrop-1000 Spectrophotometer (Labtech International, East Sussex, UK). cDNA was prepared using Bioscript-MMLV Kit (Bioscript, London, UK). qPCR was performed using KAPA SYBR FAST qPCR Kit (Kapa Biosystems Inc., Wilmington, MA, USA) and an Opticon quantitative PCR thermal cycler (Bio-Rad, Hemel Hempstead, UK). The CCL5 and CCL20 expression ratios of samples were calculated by normalising to the reference gene (YWHAZ). The primer sequences used were as follows: CCL5, 5′-GGGTACCATGAAGATCTCTGCA-3′ (forward) and 5′-TTGGCGGTTCCTTCGAGTGA-3′ (reverse); CCL20, 5′-AATGGCCTGCGGTGGCAA-3′ (forward) and 5′-CATCGGCCATCTGTCTTGTGA-3′ (reverse); YWHAZ, 5′-TTCTTGATCCCCAATGCTTC-3′ (forward) and 5′-TTCTTGTCATCACCAGCAGC-3′ (reverse); IL-1β, 5′-TGTAATGAAAGACGGCACACC-3′ (forward) and 5′-TCTTCTTTGGGTATTGCTTGG-3′ (reverse); IL-33, 5′-AGACCAGGTGCTACTACGCTAC-3′ (forward) and 5′-CACCATCAGCTTCTTCCCATCC-3′ (reverse).

### Primary culture of mouse small intestinal crypt organoids

Organoids were prepared using published protocols.^42^ Briefly, fragments of small intestine were transferred into crypt isolation buffer (2 mM ethylenediaminetetraacetic acid in PBS) under gentle agitation at 4 °C for 30 min. The released crypts were resuspended in Matrigel (BD) (300–500 crypts per 50 ml) and cultured in advanced Dulbecco's modified Eagle's medium/F12+GlutaMAX (Life Technologies) supplemented with 10 mM HEPES (Sigma-Aldrich), 1% penicillin/streptomycin (Sigma-Aldrich), 1% N2 supplement (Life Technologies), 2% B27 supplement (Life Technologies), murine recombinant epidermal growth factor (50 ng ml^−1^), murine recombinant Noggin (100 ng ml^−1^) (PeproTech EC, London, UK) and human recombinant R-spondin1 (1 μg ml^−1^) (R&D Systems). Well-differentiated crypt organoids were split into 30 organoids per 50 μl Matrigel/well and used for subsequent experiments.

### Generation of bone marrow chimeras

Donor mice were killed for collecting bone marrow. Bone marrow cells collected were counted and resuspended in sterile PBS. After irradiation, the recipient mice were inoculated intravenously via the tail vein with 5 million donor cells in a volume of 200 μl. Recipient mice were given mash to eat with antibiotic water containing 2 mg ml^−1^ of neomycin sulphate (Sigma-Aldrich) and used for experiments after 6 weeks of bone marrow reconstitution.

### Statistics

Statistical analyses were performed using Student's *t*-test, and one- or two-way ANOVA. The *P*-values of <0.05 were considered significant. All statistical analyses were carried out using GraphPad Prism for windows, version 5 (GraphPad, La Jolla, CA, USA).
