# Supplementary material for: P2X7 receptor-dependent tuning of gut epithelial responses to infection
Source: Immunol Cell Biol. 2016 Sep 20;95(2):178–88. doi: 10.1038/icb.2016.75 (PMC5181772; doi:10.1038/icb.2016.75)
Supplement: Supplementary Information [file icb201675x1.pdf]

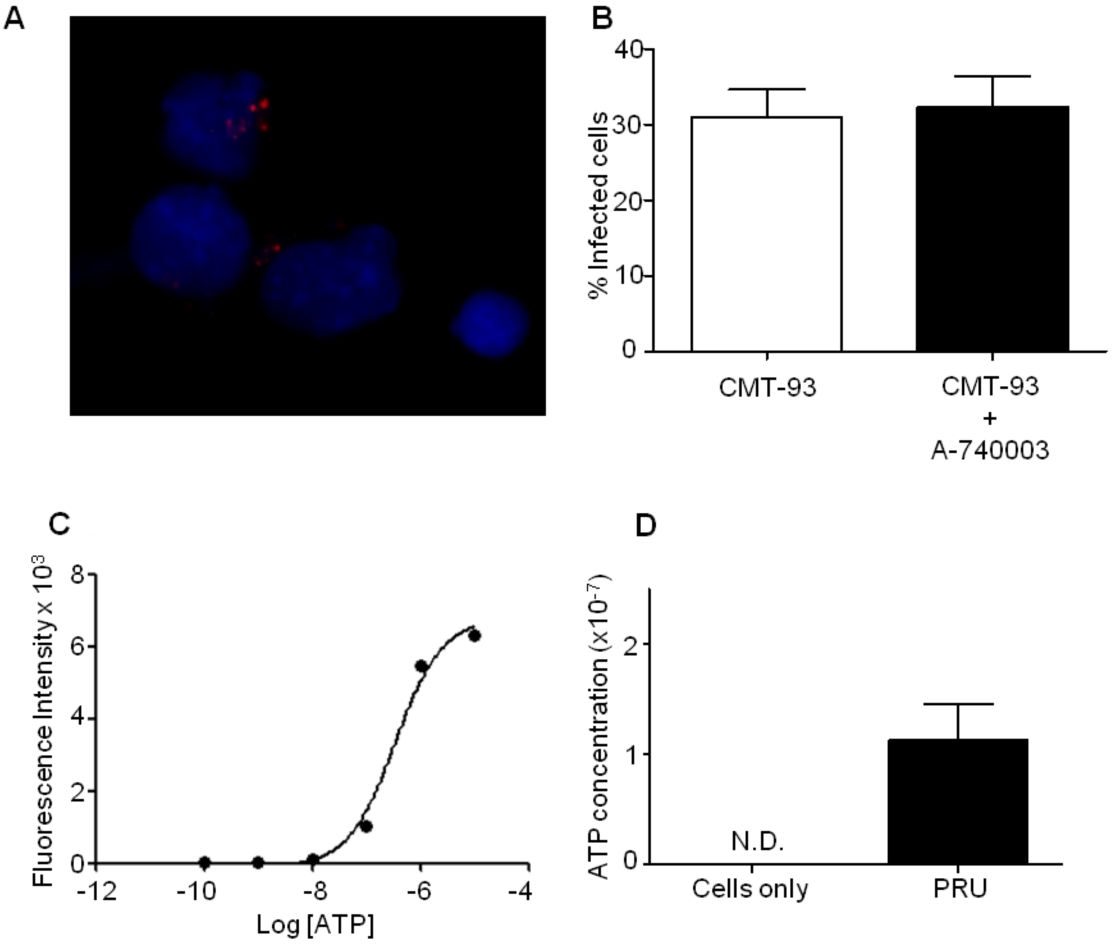

Supplementary Figure 1. Toxoplasma infection induces ATP section by gut epithelial cells. Epithelial cells were infected with *T. gondii* PRU strain in a ratio of 1:1 for 6hrs and infectivity in the presence or absence of the P2X7R antagonist assessed by (**A**) microscopy and enumerated by (**B**) flow cytometry. The concentrations of ATP in the supernatants after 24 hours of infection were measured using the luciferin–luciferase bioluminescence assay. Shown is (**C**) the absorbance curve of ATP and (**D**) the ATP concentration data of control versus infected cells. Data shown are means ± SD (n=3 per group). N.D.: non-detectable.

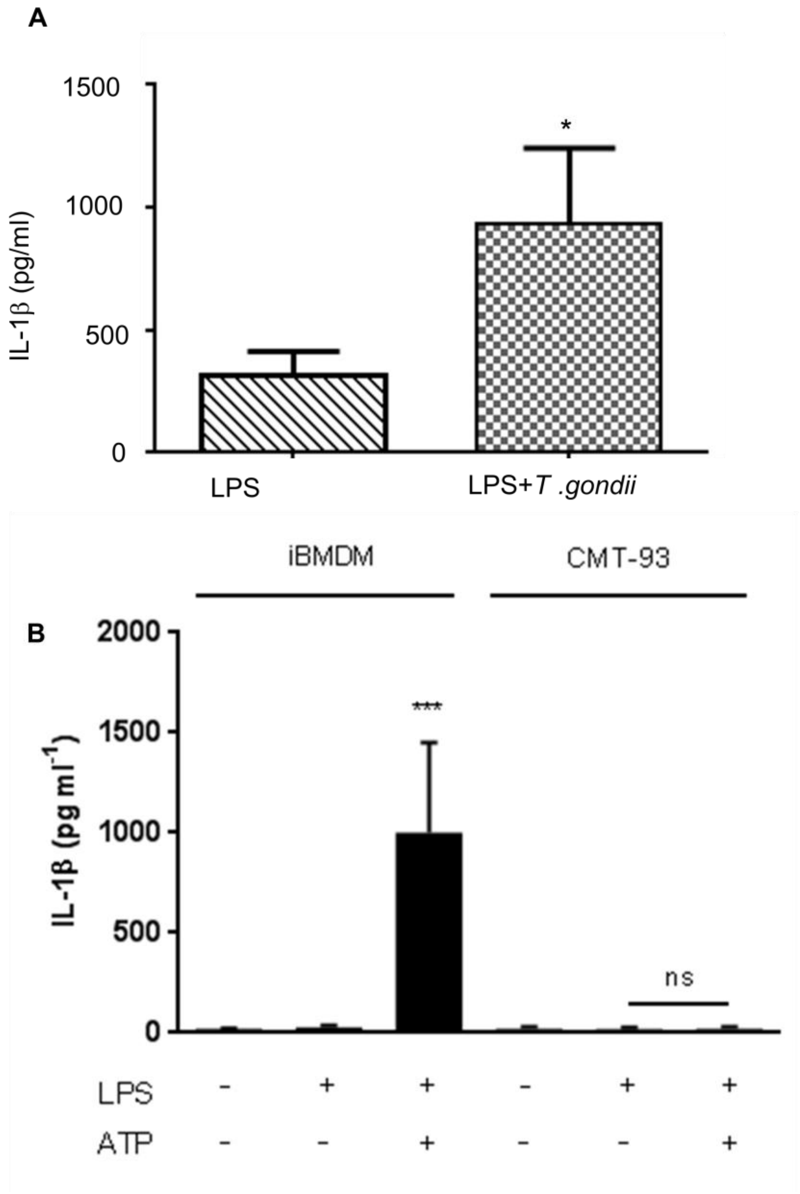

Supplementary Figure 2 Comparative analyses of macrophage response to infection stimuli

(A) IL1β secretion by LPS-primed Thp-1 monocyte cells was significantly increased following infection with *T. gondii* (n=3-4) (B) Murine immortalised bone marrow-derived macrophages (iBMDMs) and epithelial CMT-93 cells were primed with LPS (1µg/mL) and stimulated with 5 mM ATP and IL-1β analyzed by ELISA. Data are presented as mean + s.e.m from 4 independent experiments. ***P<0.001 vs iBMDMs treated with LPS alone or CMT-93 cells treated with LPS and ATP.

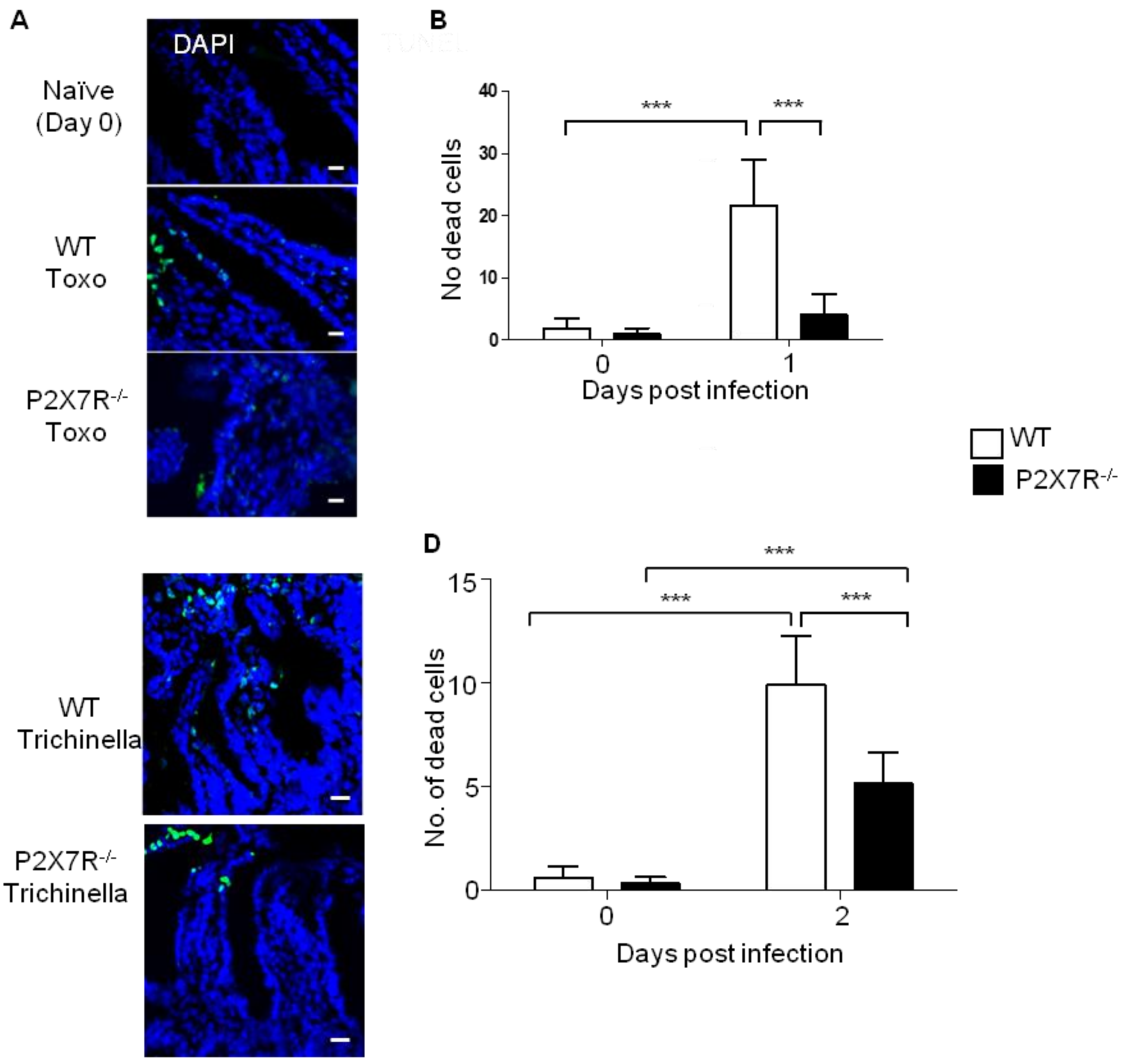

Supplementary Figure 3: Reduced apoptosis in vivo in the absence of p2X7R

(**A**) Representative images of TUNEL stained sections of ileum from WT and P2X7R$^{-/-}$ mice day 0 and day 1 p.i. with *T. gondii* with the TUNEL^{+} dead cells in green. Sections were counterstained with DAPI (blue). (**B**) Quantification of cell death (TUNEL^{+} cells) in WT (white bars) and P2X7R-/- mice (black bars). (**C**) Representative images of TUNEL stained sections from WT and P2X7R$^{-/-}$ mice day 2 p.i. with *T. spiralis* and (D) the corresponding quantification. n= 6 per group, pooled from two independent experiments (means ± SD). Statistical difference was measured using two-way ANOVA. ***$P < 0.001$.

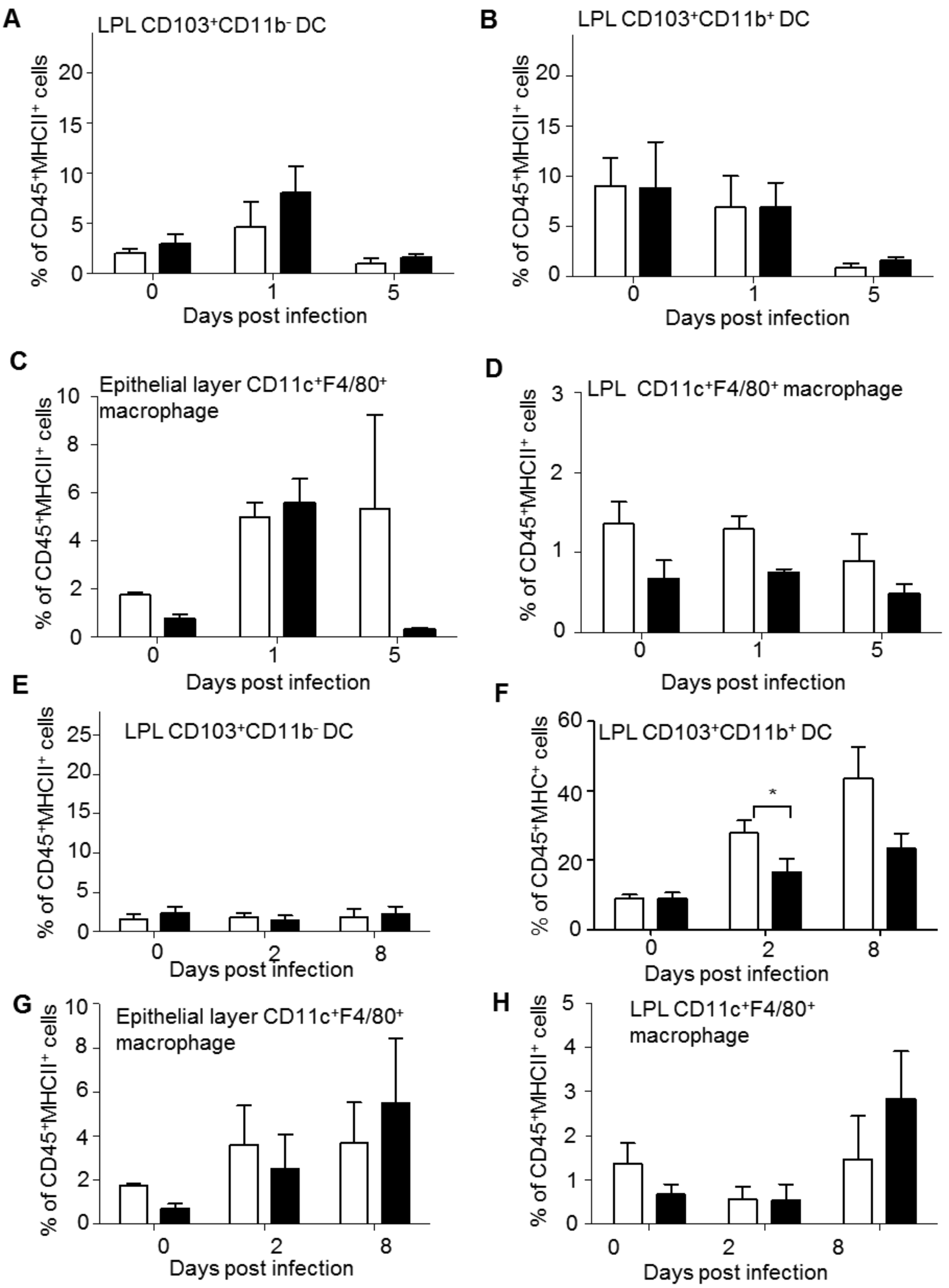

Supplementary Figure 4. P2X7R deficiency does not affect the recruitment of small intestinal CD103$^-$ DCs and F4/80$^+$CD11c$^+$ macrophages in response to infection. C57BL/6 (open bars) and P2X7R$^{-/-}$ mice (black bars) were orally infected with *T. gondii* (1x10^6 tachyzoites/mouse) and sacrificed at Day 0 and 1 p.i.. Small intestinal IEC and LPL cells from *T. gondii*-infected mice were

collected and analysed by flow cytometry. The frequency of small intestinal (**A**) epithelial layer and (**B**) LPL compartment of $CD45^{+}MHCII^{+}F4/80^{-}CD103^{-}CD11b^{+}$ DC ($CD103^{-}CD11b^{+}$ DC) and (**C**) epithelial layer and (**D**) LPL compartment of $CD45^{+}MHCII^{+}CD11b^{+}CD11c^{+}F4/80^{+}$ macrophage ($CD11c^{+}F4/80^{+}$ macrophage) were calculated as the percentage of $CD45^{+}MHCII^{+}$ cell population. n= 6 per group, pooled from two independent experiments (means ± SD). **P*< 0.05 and ****P*< 0.001 as compared with other time-point groups. Statistical difference was measured using two-way ANOVA with Bonferroni post test.
